# Catheter ablation of concomitant atrial fibrillation improves survival of patients undergoing transcatheter edge-to-edge mitral valve repair

**DOI:** 10.3389/fcvm.2023.1229651

**Published:** 2023-08-14

**Authors:** Felix Ausbuettel, Sebastian Barth, Georgios Chatzis, Dieter Fischer, Sebastian Kerber, Julian Mueller, Stephan List, Philipp Halbfass, Thomas Deneke, Holger Nef, Hans-Helge Mueller, Dimitar Divchev, Bernhard Schieffer, Ulrich Luesebrink, Christian Waechter

**Affiliations:** ^1^ Department of Cardiology, University Hospital Marburg, Philipps University Marburg, Marburg, Germany; ^2^ Department of Cardiology, Cardiovascular Center Bad Neustadt/Saale, Bad Neustadt an der Saale, Germany; ^3^ Department of Cardiology, Cardiovascular Center Rotenburg/Fulda, Rotenburg an der Fulda, Germany; ^4^ Department of Cardiology, University Hospital Oldenburg, Carl von Ossietzky University Oldenburg, Oldenburg, Germany; ^5^ Department of Cardiology, University Hospital Gießen, Justus Liebig University Gießen, Gießen, Germany; ^6^ Institute for Bioinformatics and Biostatistics, Philipps University Marburg, Marburg, Germany

**Keywords:** pulmonary vein isolation, atrial fibrillation, mortality, heart failure, mitraclip, PASCAL, mitral regurgitation, catheter ablation

## Abstract

**Background:**

Atrial fibrillation (AF) is the most common concomitant disease in patients undergoing transcatheter edge-to-edge repair (TEER) for mitral regurgitation (MR) and detrimentally affects their outcome. While there is increasing evidence for prognostic improvement and safety of catheter ablation (CA) of AF in the overall cohort of heart failure patients, corresponding data in TEER patients are lacking.

**Objectives:**

To investigate the impact of treatment regimens for concomitant AF on survival of TEER patients.

**Methods:**

In a multicenter observational cohort study consecutive patients successfully undergoing TEER were analyzed and survival of patients receiving CA of concomitant AF was compared with that of patients on pharmacological AF treatment and with that of patients without a history of AF, using propensity score matching (PSM).

**Results:**

A total of 821 patients were analyzed. Of these, 608 (74.1%) had concomitant AF, of whom 48 patients received CA. Patients with CA in AF showed significantly higher 3-year-survival after TEER compared to PSM-patients on pharmacological AF treatment (75.5% [36/48] vs. 49.4% [166/336], *p* = 0.009). The 3-year-survival after TEER of patients with concomitant AF treated with CA was not significantly different from PSM-patients without AF (75.5% [36/48] vs. 68.3% [98/144], *p* = 0.36).

**Conclusions:**

CA of AF is superior to pharmacotherapy as it significantly improves the survival of TEER patients in a PSM analysis. CA even offsets the prognostic disadvantage of coexisting AF in TEER patients. Given the growing evidence of prognostic benefits in the overall cohort of HF patients, our data point out the importance of treating concomitant AF and support CA as an essential part of a holistic management of TEER patients.

## Graphical Abstract

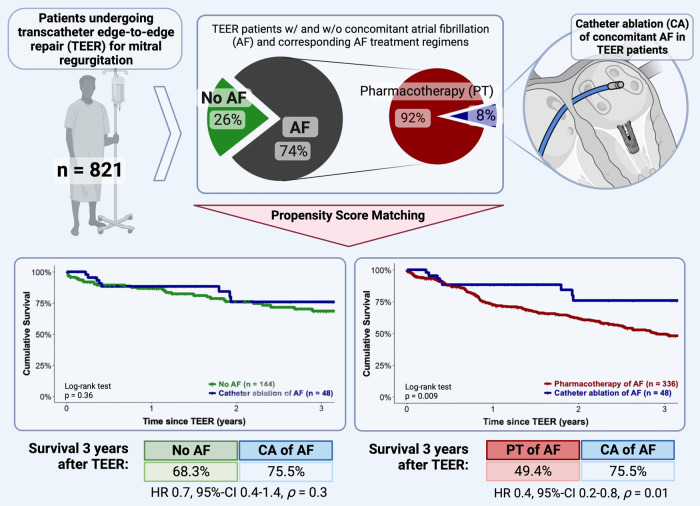

## Introduction

Mitral regurgitation (MR) represents one of the most common valvular heart diseases (VHD) in developed and industrialized countries. Especially in the elderly population over 75 years of age, it is the predominant form of VHD, with a prevalence of about 10%, and a major contributor to the development of heart failure (HF) (1). According to the projected demographic trends of aging societies, the number of these patients will increase dramatically in the decennia to come (2). However, already today as many as half of all patients with MR requiring therapy are not treated surgically due to advanced HF and multiple comorbidities associated with an unacceptably high perioperative risk (3, 4). For this particular high-risk cohort, transcatheter edge-to-edge repair (TEER) has proven to be a safe and beneficial treatment modality, alleviating HF symptoms (5) and even improving prognosis in selected MR etiologies (6, 7). With a prevalence of up to 75.4% (8), atrial fibrillation (AF) is the most common comorbidity in TEER collectives and, as demonstrated in a multitude of recent studies, dramatically worsens the medium- and long-term outcome of successfully treated TEER patients (9–13). In contrast to the collective of surgically treated MR patients, in whom there is convincing evidence of prognostically favorable concomitant rhythm control of AF (14), reflected in a class I-A recommendation in the relevant guideline (15), the evidence in TEER patients regarding the prognostic impact of AF treatment regimens is limited.

We recently demonstrated that the majority of TEER patients with coexistent AF were on rate control therapy, which was associated with a more favorable long-term outcome than a pharmacological rhythm control (13, 16). Data on the prognostic impact of state-of-the-art interventional rhythm control in this unique patient population, however, are lacking. Using the established statistical methods of propensity score matching (PSM) and multivariable Cox regression, we analyze the prognostic effect of catheter ablation (CA) of concomitant AF in comparison to pharmacological AF management in a well-characterized multicenter collective of TEER patients.

## Materials and methods

### Data collection and definitions

Data on all consecutive patients scheduled for TEER after a consensus decision by a multidisciplinary heart team at four tertiary cardiac centers in Germany between October 2011 and December 2022 were recorded in registries of the participating center and subsequently pooled for the present analysis. Eligibility for TEER was defined according to relevant guidelines (17): generally, patients with severe symptomatic primary MR, and patients with secondary MR who remain highly symptomatic despite guideline-guided treatment of heart failure, who are at high or prohibitive risk for surgery, and who meet echocardiographic eligibility criteria for TEER. Here, the multidisciplinary heart teams mainly oriented on the echocardiographic criteria defined in the Endovascular Valve Edge-to-Edge Repair Study (EVEREST) II for primary MR and on those defined in the Cardiovascular Outcomes Assessment of the MitraClip Percutaneous Therapy for Heart Failure Patients with Functional Mitral Regurgitation (COAPT) trial for secondary MR (5, 6). Regarding procedural outcome, successful TEER was defined as MR reduction to less than or equal to moderate severity and a pressure gradient across the mitral valve of 5 mmHg or less after clip implantation. The definition of AF types and therapies and procedural aspects were as defined in recent publications (13, 16), which also addressed how patient selection was carried out. In brief, the definition of AF types was made according to the recent guidelines of the European Society of Cardiology (18). Correspondingly, paroxysmal AF was defined as lasting seven days or less. All AF episodes exceeding seven days were defined as persistent AF, presuming that a rhythm control strategy was still being pursued. Permanent atrial fibrillation was defined as when rhythm-controlling measures were no longer performed by mutual agreement between the patient and the treating physician.

All patients with non-permanent AF who underwent catheter ablation (CA) of AF within 24 months before or after the TEER procedure were identified and assigned to the interventional rhythm control group. The ablation procedure always included antral isolation of the pulmonary vein *ostia*. The creation of additional lesions was left to the discretion of the individual physician. CA-associated major complications were defined as access site bleeding or hematoma requiring blood transfusion or surgical intervention, pericardial effusion requiring pericardiocentesis, systemic and/or cerebral embolization, phrenic nerve palsy, pulmonary vein stenosis, esophageal ulceration and/or fistula or periprocedural mortality. All remaining AF patients were assigned to the pharmacological AF treatment group. Furthermore, all patients with non-permanent AF treated with antiarrhythmic drugs (AAD) of Vaughan-Williams classes I-IV, or a combination of these were defined to be on pharmacological rhythm control therapy. The indication for the use of AAD, especially that for amiodarone (class III AAD), was carefully considered in each case to exclude indications other than rhythm control in AF (e.g., ventricular arrhythmias) from further analysis. In accordance with the determinant decision to forgo further rhythm-maintaining therapies, all patients with permanent AF were defined to be on rate control therapy. Major adverse cardiac and cerebrovascular events (MACCE) were defined as the occurrence of a cerebral and/or systemic thromboembolic event, a hemorrhage requiring intervention and/or transfusion or in-hospital morality from a cardiovascular cause. The local ethics committee approved the study (reference number 120/18, Philipps University Marburg, Germany).

### Statistical analysis

**Figure 1:**
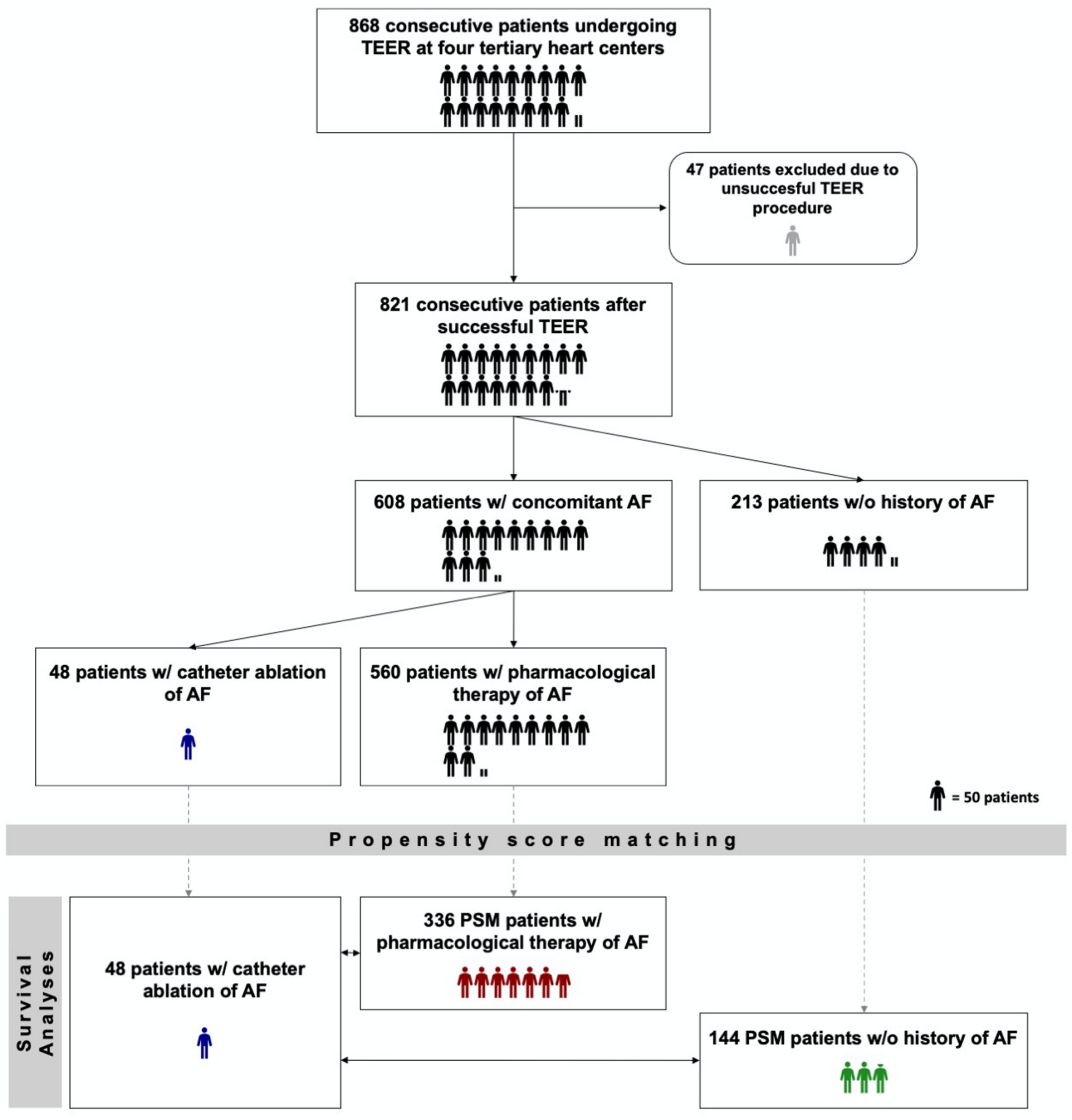
Study design flow chart. TEER, transcatheter edge-to-edge mitral valve repair; AF, atrial fibrillation; PSM, propensity score matching. w/ - with. w/o – without. *One humanoid icon corresponds to 50 patients. If the number of patients could not be divided by 50, a correspondingly proportional humanoid icon was used (e.g. one half humanoid icon corresponds to 25 patients.).*

All statistical analyses were performed by using R Studio V3.6.1 (R Foundation for Statistical Computing, Vienna, Austria), including the “survival”-, “MatchIt”-, “survminer”-, “stddiff”-, “My.Stepwise”- and “dplyr”-Packages as well as GraphPad Prism 6.0 (Dotmatics, Boston, MA, USA). Continuous variables are presented with mean and standard deviation for normally distributed variables and with median and interquartile ranges (IQR: 25th–75th percentile) for non-normally distributed variables. Categorical variables are presented as frequencies and percentages (%). Differences between two groups were compared for categorical variables with the chi-square test when the expected cell size was ≥20 and with Fisher's exact test when the expected cell size in one or more cells was <20. For continuous variables, Student's t test was used for normally distributed variables and Wilcoxon's test was used for non-normally distributed variables. The normal distribution of continuous variables was validated with the Shapiro-Wilk test. A two-tailed *p*-value of <0.05 was considered statistically significant. To address differences in baseline characteristics and to achieve the most unbiased comparison possible between outcomes of patients with interventional and pharmacological rhythm control, with rate control, and of patients without a history for AF, propensity score matching (PSM) analysis was performed using the nearest neighbor matching with a caliper width set at 0.2 standardized difference of the logit of the estimated propensity scores. In order to include as many subjects as possible from the total cohort in the analyses, the groups to be studied were matched at different ratios. The matching ratio was based on the group with the smallest number of patients, the interventional rhythm control group, and on ensuring a sufficient balance of baseline characteristics. With regard to appropriate matching parameters, we used statistically significantly different (*p*-value cut off 0.05) parameters, in the corresponding baseline characteristics and previously published and generally accepted mortality predictors and mortality predictors revealed or confirmed by univariable and multivariable Cox regression analyses in the present collective. The selected matching parameters were age, coronary artery disease, pre-existing cardiac resynchronization therapy, chronic obstructive pulmonary disease, New York Heart Association (NYHA) functional class IV, angiotensin receptor-neprilysin inhibitor usage, STS risk score as well as concomitant severe tricuspid regurgitation. Following matching, time-to-event analysis for the different AF treatment strategies was conducted according to the Kaplan-Meier method; differences between groups were compared using the log-rank test. Both univariable and multivariable Cox regression were performed to determine independent predictors of mortality. Variables with *p* < 0.1 in the univariate analysis were included in the multivariable Cox regression model. The primary end point in the survival analyses was death from any cause. Figure 1 provides a flowchart of the study design.

### Missing data

In cases where follow-up data were insufficient, they were supplemented by a survival query to the registry office for patients lost to follow-up. In spite of all efforts, 55 patients (6.7%) could not be followed-up on during the indicated study period because of an unreported change of residence. There was no evidence of informative missingness and no significant impact of “lost to follow-up” patients on the results presented. Supplementary Table S1 provides baseline characteristics of patients lost to follow-up compared with the entire cohort studied.

## Results

During the study period, 868 patients undergoing TEER for severe MR were identified at the four participating centers. For the TEER procedure, the MitraClip® device (Abbott Vascular, Santa Clara, CA, USA) was used in 650 (79.2%) and the PASCAL™ system (Edwards Lifescience, Irvine, CA, USA) in 171 (20.8%) of cases. Forty-seven patients (5.4%) underwent conservative treatment or surgical intervention due to insufficient reduction in MR severity and were therefore excluded from further analysis. The type of TEER device used had no significant influence on the success or safety of the procedure (Odds ratio [OR] 0.9, 95%-confidence interval [CI] 0.5–1.4, *p* = 0.6 and OR 1.1, 95%-CI 0.5–2.2, *p* = 0.8, respectively). Concomitant AF or underlying AF treatment regimen also did not affect the risk of unsuccessful TEER intervention, as reported previously (13, 16). The well-established association of concomitant AF with adverse outcomes after successful TEER was also confirmed in the studied collective [Hazard ratio (HR) 1.3, 95%-CI 1.03–1.7, *p* = 0.046].

Clinical and procedural characteristics of the entire cohort are presented in Table 1 (first column). The median follow-up time was 397 days (IQR 890 days).

**Table 1:** Clinical and procedural characteristics of the overall cohort studied and of patients with catheter ablation of concomitant AF and of patients with pharmacological AF therapy before and after propensity score matching at the time of the TEER procedure.

Variable		Before propensity score matching			After propensity-score-matching		
	All patients	Catheter ablation of AF	Pharmacological AF therapy	*p*-value	Catheter ablation of AF	Pharmacological AF therapy	*p*-value
	(*n* = 821)	(*n* = 48)	(*n* = 560)		(*n* = 48)	(*n* = 336)	
Age (years)	78 ± 8	74 ± 9	79 ± 7	**<0**.**001**	74 ± 9	76 ± 8	0.07
euroSCORE II (IQR)^a^	16.0 (19.9)	17.0 (15.8)	15.0 (21.9)	0.8	17.0 (15.8)	14.0 (19)	0.8
STS risk score (IQR)^a^	6.6 (8)	6.3 (4.5)	7.0 (8.6)	0.1	6.3 (4.5)	6.0 (7.7)	0.7
Male sex	62.6% (514)	70.8% (34)	63.7% (357)	0.4	70.8% (34)	65.8% (221)	0.5
NYHA class I	0.1% (1)	0% (0)	0.2% (1)	0.4	0% (0)	0.3% (1)	0.6
NYHA class II	3.2% (26)	0% (0)	3.0% (17)		0% (0)	3.6% (12)	
NYHA class III	75.6% (621)	83.3% (40)	74.3% (416)		83.3% (40)	80.7% (271)	
NYHA class IV	21.1% (173)	16.8% (8)	22.5% (126)		16.8% (8)	15.5% (52)	
COPD	17.8% (146)	8.3% (4)	17.7% (99)	0.1	8.3% (4)	7.4% (25)	0.8
CAD	62.2% (511)	60.4% (29)	60.5% (339)	1	60.4% (29)	59.5% (200)	1
Prior CABG	27.8% (228)	37.5% (18)	24.6% (138)	0.06	37.5% (18)	25.6% (86)	0.09
Prior PCI	54.0% (443)	41.7% (20)	53.6% (300)	0.1	41.7% (20)	54.8% (184)	0.09
Pre-existing pacemaker	29.5% (242)	41.7% (20)	32.7% (183)	0.2	41.7% (20)	37.2% (125)	0.6
Pre-existing ICD	22.3% (183)	31.2% (15)	22.3% (125)	0.2	31.2% (15)	27.7% (93)	0.6
Pre-existing CRT	12.9% (106)	29.2% (14)	14.8% (83)	**0**.**02**	29.2% (14)	*19.6%* (*66)*	*0*.*1*
Diabetes mellitus	29.8% (245)	18.8% (9)	30.4% (170)	0.1	18.8% (9)	31.0% (104)	0.1
Arterial hypertension	81.0% (665)	83.3% (40)	82.7% (463)	1	83.3% (40)	79.5% (267)	0.7
Prior stroke	9.7% (80)	6.2% (3)	10.9% (61)	0.5	6.2% (3)	11.6% (39)	0.3
LVEF ≥50%	39.2% (322)	39.6% (19)	41.2% (231)	0.4	39.6% (19)	37.5% (126)	0.8
LVEF 41–49%	11.6% (95)	6.2% (3)	12.9% (72)		6.2% (3)	9.8% (33)	
LVEF ≤40%	49.2% (404)	54.2% (26)	45.9% (257)		54.2% (26)	52.7% (177)	
Atrial fibrillation	74.1% (608)	100% (48)	100% (560)	0.1	100% (48)	100% (336)	0.08
* Paroxysmal AF*	*21.6% (177)*	*12.5% (6)*	*30.5%* *(171)*		*12.5%* *(6)*	*31.2%* *(105)*	
* Non-paroxysmal AF*	*52.5% (431)*	*87.5% (42)*	*69.5%* *(389)*		*87.5%* *(42)*	*68.8%* *(231)*	
GFR (ml/Min)	50.0 ± 26	49.5 ± 21	49 ± 26	0.8	49.5 ± 21	48 ± 21	0.7
NT-proBNP (ng/L)^a^	2,664 (5,026)	2,466 (4,050)	2,278 (4,907)	1	2,466 (4,050)	2,996 (4,991)	0.8
TR grade III	18.6% (153)	16.8% (8)	21.8% (122)	0.5	16.8% (8)	18.5% (62)	0.8
Degenerative MR etiology	35.7% (293)	41.7% (20)	35.2% (197)	0.5	41.7% (20)	34.2% (115)	0.5
Functional MR etiology	52.6% (432)	50.0% (24)	51.8% (290)		50.0% (24)	52.4% (176)	
Mixed MR etiology	11.7% (96)	8.3% (4)	13.0% (73)		8.3% (4)	13.4% (45)	
Median procedure duration (min)^a^	80 (60)	82 (57)	80 (60)	0.8	82 (57)	80 (61)	0.8
Number of clips implanted^a^	1 (1)	1 (1)	1 (1)	0.9	1 (1)	1 (1)	0.8
Periprocedural MR reduction^b^	Δ2.0 ± 0.6	Δ2.1 ± 0.6	Δ2.1 ± 0.6	0.6	Δ2.1 ± 0.6	Δ2.1 ± 0.5	0.8
Postprocedural MR grade							
≤*mild-to-moderate*	88.6% (727)	89.6% (43)	89.6% (502)	0.9	89.6% (43)	89.5% (301)	0.9
*moderate*	11.4% (94)	10.4% (5)	10.4% (58)		10.4% (5)	10.4% (35)	
TEER device							
*MitraClip^©^*	79.2% (650)	*60.4%* *(29)*	*81.8%* *(458)*	**0**.**002**	*60.4%* *(29)*	*78.9%* *(265)*	**0**.**01**
*PASCAL™*	20.8% (171)	*39.6%* *(19)*	*18.2%* *(102)*		*39.6%* *(19)*	*21.2%* *(71)*	
Length of hospital stay (days)^a^	6 (5)	6 (3)	6 (5)	0.7	6 (3)	6 (5)	1
Periprocedural overall MACCE	5.4% (44)	4.2% (2)	5.0% (28)	1	4.2% (2)	3.6% (12)	0.7
*Cerebral/systemic thromboembolic event*	*0.6%* *(5)*	*0%* *(0)*	*0.7%* *(4)*	*1*	*0%* *(0)*	*0.3%* *(1)*	*1*
*Bleeding requiring intervention*	*3.2%* *(26)*	*4.2%* *(2)*	*2.9%* *(16)*	*0.6*	*4.2%* *(2)*	*2.4%* *(8)*	*0.4*
*In-hospital death from cardiovasc. cause*	*2.2%* *(18)*	*0%* *(0)*	*2.3%* *(13)*	*0.6*	*0%* *(0)*	*1.5%* *(5)*	*1*
In-hospital death from any cause	3.5% (29)	0% (0)	3.6% (20)	0.4	0% (0)	2.1% (7)	0.6
Heart failure therapy							
ACE-/AT1 inhibitors	72.2% (593)	58.3% (28)	73.4% (411)	**0**.**03**	58.3% (28)	69.9% (235)	0.1
ARN inhibitor	13.5% (111)	31.2% (15)	12.1% (68)	**<0**.**001**	31.2% (15)	19% (64)	0.06
Beta blockers	88.8% (729)	93.9% (45)	88.8% (497)	0.5	93.9% (45)	90.2% (303)	0.6
Diuretics	92.8% (762)	93.9% (45)	84.8% (475)	0.1	93.9% (45)	85.4% (287)	0.2
Aldosteron antagonists	48.2% (396)	58.3% (28)	48.0% (269)	0.2	58.3% (28)	50.9% (171)	0.4
SGLT-II inhibitors	4.8% (39)	10.4% (5)	3.8% (21)	**0**.**046**	10.4% (5)	6% (20)	0.2
Vericiguat	0.1% (1)	0% (0)	0.2% (1)	1	0% (0)	0.3% (1)	1
Atrial fibrillation therapy							
Oral anticoagulants	73.0% (599)	95.8% (46)	92.7% (519)	0.6	95.8% (46)	94.9% (319)	1
Class-I-AAD	0.2% (2)	0% (0)	0.4% (2)	1	0% (0)	0.3% (1)	1
Class-II-AAD	53.8% (442)	77.1% (37)	67.7% (379)	0.7	77.1% (37)	67.3% (226)	0.6
Class-III-AAD	1.7% (14)	2.1% (1)	2.3% (13)	1	2.1% (1)	2.7% (9)	1
Class-II + Class-III-AAD	10.4% (85)	16.8% (8)	13.8% (77)	0.5	16.8% (8)	15.2% (51)	0.8
Class-II-AAD + Digitalis	5.0% (41)	0% (0)	7.3% (41)	0.06	0% (0)	7.7% (26)	0.06
Class-IV-AAD	0% (0)	0% (0)	0% (0)	1	0% (0)	0% (0)	1
Catheter ablation	5.8% (48)	100% (48)	–	–	100% (48)	–	–

Data presented as percentages or mean ± SD.

^a^ Data presented as median with interquartile range (IQR).

^b^ MR grade according to American Society of Echocardiography (ASE) classification.

AF, atrial fibrillation; COPD, chronic obstructive pulmonary disease; CABG, coronary artery bypass graft surgery; PCI, percutaneous coronary intervention; ICD, implantable cardioverter defibrillator; CRT, cardiac resynchronization therapy; GFR, glomerular filtration rate; LV function, left ventricular function; LA, left atrial; NT-proBNP, N-terminal pro-B-type natriuretic peptide; TR, tricuspid regurgitation; MR, mitral regurgitation; MACCE, major adverse cardiac and cerebrovascular events; AAD, antiarrhythmic drugs; ACE, angiotensin converting enzyme; AT1, angiotensin II type 1 receptor; ARN, angiotensin receptor neprylisin; SGLT-II, sodium-glucose transporter 2.

Statistically significant *p*-values are shown in bold.

### Comparison of long-term outcomes of catheter ablation vs. pharmacological therapy of concomitant atrial fibrillation in TEER patients

Within the overall collective, 48 patients were identified who received interventional rhythm control by CA of AF. CA was performed in 50% (24/48) of patients within a mean period of 487 days (±243 days) before and in the remaining half of patients (24/48) within a mean period of 246 days (±174 days) after TEER intervention. With the exception of one case (1/48), which was treated with cryoablation, CA was performed with radiofrequency (RF) energy (47/48). The mean procedure time for CA amounted 110 min (±39 min), and acute procedural success defined as achieving bidirectional block of all pulmonary veins was reached in all patients. No CA-related periprocedural major complications or interferences with the TEER device were observed. The timing of CA, whether performed before or after TEER, did not affect the long-term prognosis (HR 1.0, 95%-CI 0.97–1.002, *p* = 0.75). However, to address a potential immortal time bias (19), the follow-up period was started after completion of both the TEER and CA procedures.

Propensity score matching (PSM) was used to identify patients with pharmacological treatment (PT) of AF from the total cohort and to assign them to the CA group. To achieve a full balance of the baseline characteristics and to include as many subjects as possible in the subsequent survival analysis, a matching ratio of 1:7 was selected. Except for the type of TEER device used, a balance of all relevant baseline characteristics was thus achieved, as shown in Table 1. Despite PSM, the PASCAL device was utilized statistically significantly more often in the CA group compared to the PT group (39.6% vs. 20.3%, *p* = 0.01).

**Figure 2:**
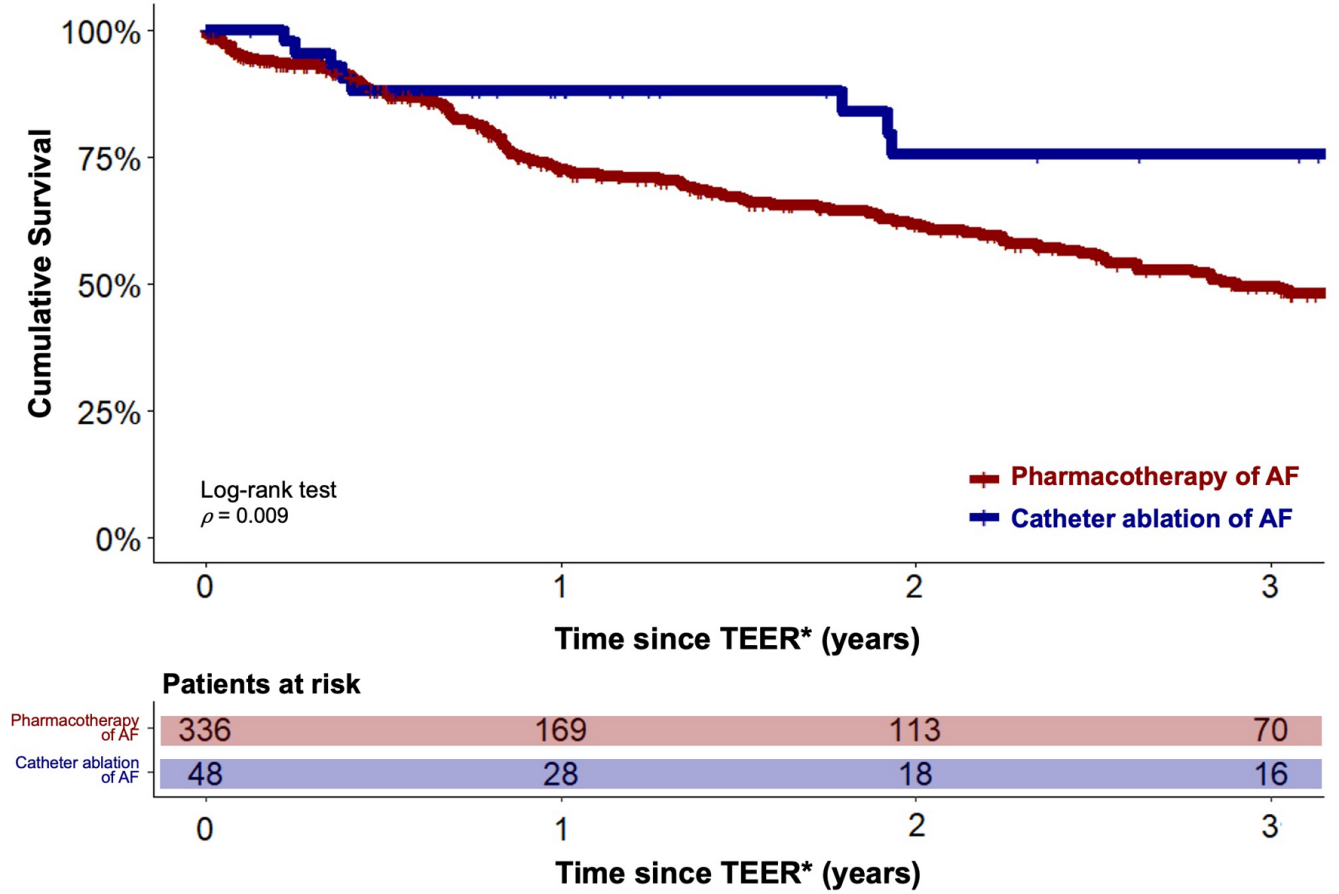
Estimated cumulative survival of TEER patients with catheter ablation and pharmacotherapy of concomitant AF. Kaplan-Meier plots showing cumulative survival of patients with catheter ablation of AF (blue graph) and of patients under pharmacotherapy of AF (red graph). Graphs indicating means and 95% confidence intervals. * - For the catheter ablation group, the follow-up period was initiated after completion of both the TEER and CA procedures.

A statistically significant reduced estimated cumulative survival of pharmacological AF treatment compared to interventional rhythm control patients was observed after 3 years (166/336 (49.4%) vs. 36/48 (75.5%), *p* = 0.009). The corresponding Kaplan-Meier diagram is plotted in Figure 2. Multivariable Cox regression revealed severe tricuspid regurgitation (TR) (HR 1.5, 95%-CI 1.3–2.2, *p* < 0.001), chronic obstructive pulmonary disease (HR 1.3, 95%-CI 1.03–1.9, *p* = 0.03) and New York Heart Association (NYHA) functional class IV (HR 1.4, 95%-CI 1.02–1.8, *p* = 0.04) to be statistically significant negative predictors of long-term survival. Whereas a statistically significant positive association was found between CA of AF and long-term outcome (HR 0.4, 95%-CI 0.2–0.8, *p* = 0.01).

For further comparison of interventional vs. pharmacological rhythm control of AF and interventional rhythm control vs. rate control of AF, multivariable Cox regression analysis was performed in the respective patient cohorts defined according to the criteria specified in the Methods section. Again, treatment of concomitant AF by CA was statistically significantly associated with better outcome in TEER patients compared with pharmacological rhythm control (HR 0.6, 95%-CI 0.3–0.9, *p* = 0.03) and rate control (HR 0.45, 95% CI 0.3–0.7, *p* = 0.006).

### Comparison of long-term outcomes of TEER patients with concomitant AF treated with catheter ablation vs. TEER patients without a history of AF

**Figure 3:**
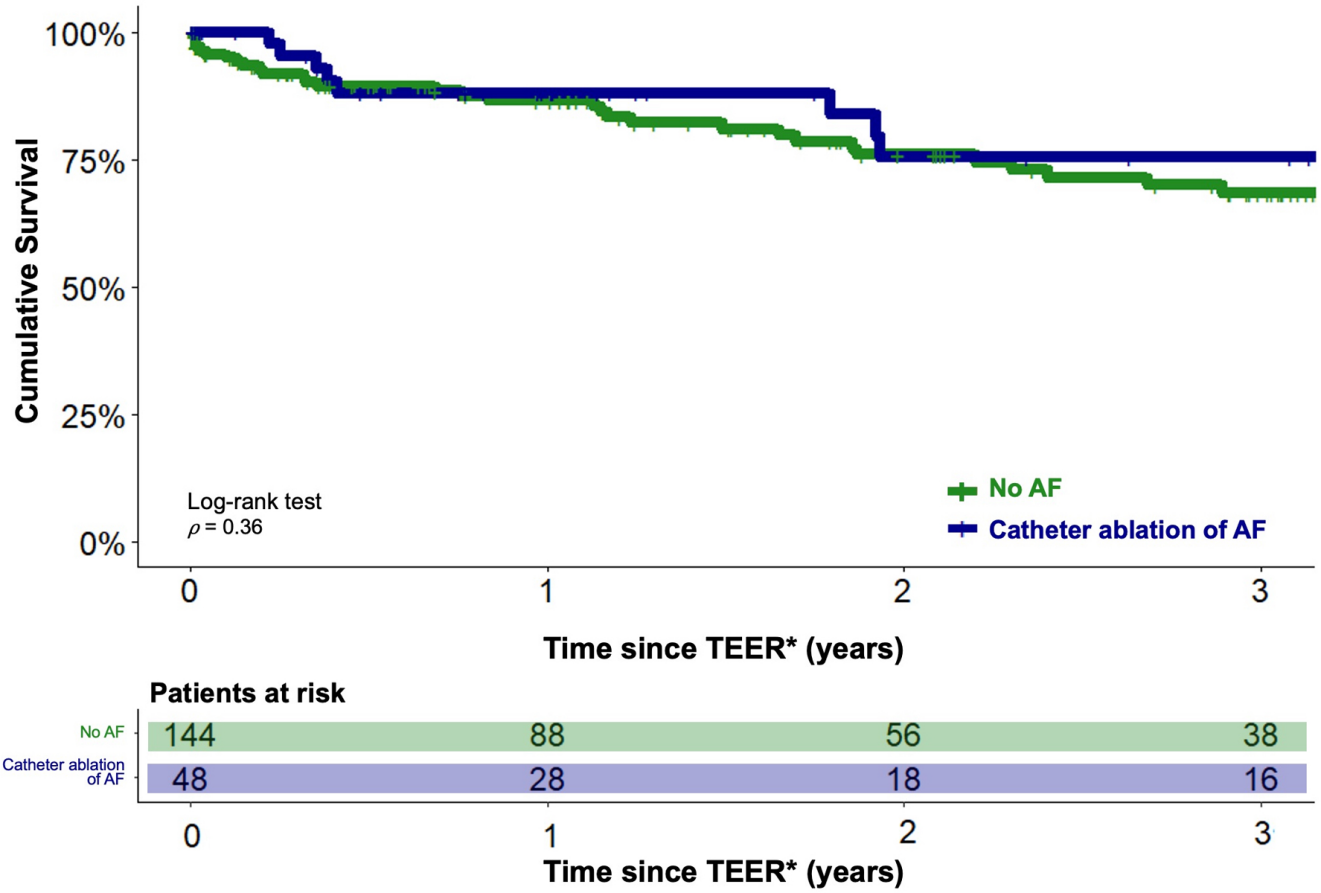
Estimated cumulative survival of TEER patients with concomitant AF treated with catheter ablation and of TEER patients without a history of AF. Kaplan-Meier plots showing cumulative survival of TEER patients with catheter ablation of concomitant AF (blue graph) and of TEER patients without a history of AF (green graph). Graphs indicating means and 95% confidence intervals. * - For the catheter ablation group, the follow-up period was initiated after completion of both the TEER and CA procedures.

Analogous to the previous analysis, the long-term outcomes of TEER patients under interventional rhythm control were compared with those of TEER patients without concomitant AF using PSM in a 1:3 ratio. Balanced characteristics are provided in Table 2. There were no statistically significant differences in the estimated cumulative survival of TEER patients with interventional rhythm-controlled AF and TEER patients without concomitant AF at 3 years after TEER procedure (98/144 (68.3%) vs. 36/48 (75.5%), *p* = 0.36). The corresponding Kaplan-Meier diagram is shown in Figure 3. Multivariable Cox regression identified NYHA functional class IV (HR 1.9, 95%-CI 1.1–3.3, *p* = 0.01) and concomitant chronic obstructive pulmonary disease (HR 2.0, 95%-CI 1.2–3.3, *p* = 0.002) to be statistically significant negative predictors of long-term survival in this comparison. CA of AF was not statistically significant associated with the long-term outcome (HR 0.8, 95%-CI 0.3–1.4, *p* = 0.3).

**Table 2:** Clinical and procedural characteristics of patients with catheter ablation of concomitant AF and of patients without a history of AF before and after propensity score matching.

Variable	Before propensity-score-matching			After propensity-score-matching		
	Catheter ablation of AF	Non-AF	*p*-value	Catheter ablation of AF	Non-AF	*p*-value
	(*n* = 48)	(*n* = 213)		(*n* = 48)	(*n* = 144)	
Age (years)	74 ± 9	77 ± 9	0.06	74 ± 9	74 ± 8	0.9
euroSCORE II (IQR)^a^	17.0 (15.8)	16.0 (18.3)	0.9	17.0 (15.8)	16.0 (16.8)	1
STS risk score (IQR)^a^	6.3 (4.5)	5.9 (7.6)	1	6.3 (4.5)	5.5 (6.4)	0.3
Male sex	70.8% (34)	57.7% (123)	0.1	70.8% (34)	59.0% (85)	0.2
NYHA class I	0% (0)	0% (0)	0.4	0% (0)	0% (0)	0.6
NYHA class II	0% (0)	4.2% (9)		0% (0)	2.8% (4)	
NYHA class III	83.3% (40)	77.5% (165)		83.3% (40)	84.7% (122)	
NYHA class IV	16.8% (8)	18.3% (39)		16.8% (8)	12.5% (18)	
COPD	8.3% (4)	20.2% (43)	0.06	8.3% (4)	10.4% (15)	0.8
CAD	60.4% (29)	67.1% (143)	0.4	60.4% (29)	66.0% (95)	0.5
Prior CABG	37.5% (18)	33.8% (72)	0.6	37.5% (18)	37.5% (54)	1
Prior PCI	41.7% (20)	57.7% (123)	0.053	41.7% (20)	57.6% (83)	0.07
Pre-existing pacemaker	41.7% (20)	18.3% (39)	**0**.**006**	41.7% (20)	23.6% (34)	0.09
Pre-existing ICD	31.2% (15)	20.2% (43)	0.1	31.2% (15)	24.3% (35)	0.3
Pre-existing CRT	29.2% (14)	12.2% (26)	**0**.**006**	29.2% (14)	16.7% (24)	0.09
Diabetes mellitus	18.8% (9)	31% (66)	0.1	18.8% (9)	30.6% (44)	0.1
Arterial hypertension	83.3% (40)	76.1% (162)	0.3	83.3% (40)	75.7% (109)	0.3
Prior stroke	6.2% (3)	7.5% (16)	1	6.2% (3)	6.9% (10)	1
LVEF ≥50%	39.6% (19)	33.8% (72)	0.7	39.6% (19)	29.2% (42)	0.3
LVEF 41–49%	6.2% (3)	9.4% (20)		6.2% (3)	11.8% (17)	
LVEF ≤40%	54.2% (26)	56.8% (121)		54.2% (26)	59.0% (85)	
Atrial fibrillation	100% (48)	0% (0)	**–**	100% (48)	0% (0)	–
* Paroxysmal AF*	*12.5%* *(6)*	–		*12.5%* (*6)*	–	
* Non-paroxysmal AF*	*87.5%* *(42)*	–		*87.5%* *(42)*	–	
GFR (ml/Min)	49.5 ± 21	54 ± 25	0.2	49.5 ± 21	54 ± 21	0.2
NT-proBNP (ng/L)^a^	2,466 (4,050)	2,069 (5,727)	0.7	2,466 (4,050)	2,430 (5,848)	0.4
TR grade III	16.8% (8)	10.8% (23)	0.3	16.8% (8)	9.7% (14)	0.2
Degenerative MR etiology	41.7% (20)	35.7% (76)	0.7	41.7% (20)	36.1% (52)	0.8
Functional MR etiology	50.0% (24)	55.4% (118)		50.0% (24)	54.9% (79)	
Mixed MR etiology	8.3% (4)	8.9% (19)		8.3% (4)	9.0% (13)	
Median procedure duration (min)^a^	82 (57)	80 (60)	0.9	82 (57)	82 (58)	0.8
Number of clips implanted^a^	1 (1)	1 (1)	0.7	1 (1)	1 (1)	0.6
Periprocedual MR reduction^b^	Δ2.1 ± 0.6	Δ2.0 ± 0.6	0.2	Δ2.1 ± 0.6	Δ2.0 ± 0.6	0.2
Postprocedural MR grade						
≤*mild-to-moderate*	89.6% (43)	85.5% (182)	0.8	89.6% (43)	84.7% (122)	0.8
*moderate*	10.4% (5)	14.5% (31)		10.4% (5)	15.3% (22)	
TEER device	* *	* *	**0**.**06**	* *	* *	0.2
* MitraClip^©^*	*60.4%* *(29)*	*76.5%* *(163)*		*60.4%* *(29)*	*72.2%* *(104)*	
* PASCAL™*	*36.9%* *(19)*	*23.5%* *(50)*		*39.6%* *(19)*	*27.8%* *(40)*	
Length of hospital stay (days)^a^	6 (3)	6 (4)	0.8	6 (3)	6.5 (4)	0.8
Periprocedural overall MACCE	4.2% (2)	6.6% (14)	0.7	4.2% (2)	6.2% (9)	0.7
* Cerebral/systemic thromboembolic event*	*0%* *(0)*	*0.5%* *(1)*	*1*	*0%* *(0)*	*0%* *(0)*	*1*
* Bleeding requiring intervention*	*4.2%* *(2)*	*3.8%* *(8)*	*1*	*4.2%* *(2)*	*3.5%* *(5)*	*1*
* In-hospital death from cardiovasc. cause*	*0%* *(0)*	*2.3%* *(5)*	*0.6*	*0%* *(0)*	*2.8%* *(4)*	*0.6*
In-hospital death from any cause	0% (0)	4.2% (9)	0.4	0% (0)	4.2% (6)	0.3
Heart failure therapy						
ACE-/AT1 inhibitors	58.3% (28)	72.3% (154)	0.08	58.3% (28)	69.4% (100)	0.2
ARN inhibitor	31.2% (15)	13.1% (28)	**0**.**004**	31.2% (15)	18.1% (26)	0.07
Beta blockers	93.9% (45)	87.8% (187)	0.3	93.9% (45)	88.9% (128)	0.4
Diuretics	93.9% (45)	90.1% (192)	0.6	93.9% (45)	87.5% (126)	0.3
Aldosteron antagonists	58.3% (28)	46.5% (99)	0.2	58.3% (28)	48.6% (70)	0.3
SGLT-II inhibitors	10.4% (5)	6.1% (13)	0.3	10.4% (5)	6.9% (10)	0.5
Vericiguat	0% (0)	0% (0)	1	0% (0)	0% (0)	1
Atrial fibrillation therapy						
Oral anticoagulants for AF	95.8% (46)	–	–	95.8% (46)	–	–
Class-I-AAD	0% (0)	–	–	0% (0)	–	–
Class-II-AAD	77.1% (37)	–	–	77.1% (37)	–	–
Class-III-AAD	2.1% (1)	–	–	2.1% (1)	–	–
Class-II + Class-III-AAD	16.8% (8)	–	–	16.8% (8)	–	–
Class-II-AAD + Digitalis	0% (0)	–	–	0% (0)	–	–
Class-IV-AAD	0% (0)	–	–	0% (0)	–	–
Catheter ablation	100% (48)	–	–	100% (48)	–	–

Data presented as percentages or mean ± SD.

^a^ Data presented as median with interquartile range (IQR).

^b^ MR grade according to American Society of Echocardiography (ASE) classification.

AF, atrial fibrillation; COPD, chronic obstructive pulmonary disease; CABG, coronary artery bypass graft surgery; PCI, percutaneous coronary intervention; ICD, implantable cardioverter defibrillator; CRT, cardiac resynchronization therapy; GFR, glomerular filtration rate; LV function, left ventricular function; LA, left atrial; NT-proBNP, N-terminal pro-B-type natriuretic peptide; TR, tricuspid regurgitation; MR, mitral regurgitation; MACCE, major adverse cardiac and cerebrovascular events; AAD, antiarrhythmic drugs; ACE, angiotensin converting enzyme; AT1, angiotensin II type 1 receptor; ARN, angiotensin receptor neprylisin; SGLT-II, sodium-glucose transporter 2.

Statistically significant *p*-values are shown in bold.

## Discussion

Reflecting the complex and intimate pathophysiological interactions, AF represents the most common concomitant disease in severe MR and has been shown to dramatically worsen the prognosis of patients undergoing TEER. This underscores the urgent need for additional strategies to improve the outcomes in this unique HF patient population. Addressing this highly relevant but still unmet clinical need, we demonstrate in a large, well-characterized multicenter collective that an interventional strategy for rhythm control of AF, as opposed to pharmacological rhythm control or rate control, is associated with a more favorable long-term outcome in TEER patients. Here, interventional rhythm control even offsets the prognostic disadvantage of coexisting AF, as the outcome is not significantly different from that of TEER patients without a history of AF. The present key findings parallel evidence derived from patients undergoing surgery for MR, which also demonstrate a beneficial impact of surgical ablation (SA) of concomitant AF as part of the valve procedure. Thus, multiple studies show that SA can be safely added to the intended surgical intervention and that it improves the long-term survival considerably compared with conservatively managed or untreated preoperative AF, which is subsequently not even significantly different form that of patients without AF (20–23). This body of evidence led to a class I recommendation (level of evidence A) for SA of AF with concomitant mitral valve surgery in the 2017 STS issued “Clinical Practice Guidelines in Surgical Treatment of Atrial Fibrillation” (15). Of course, it must also be recognized that due to the nature of TEER, these patients differ significantly from surgical collectives in terms of HF severity and degree of multimorbidity. In further agreement with the present results, substantial evidence of a prognostically favorable effect of CA of AF has also emerged in recent years for the overall collective of HF patients. Thus, the “Ablation Versus Amiodarone for Treatment of Persistent Atrial Fibrillation in Patients With Congestive Heart Failure and an Implanted Device” (AATAC) randomized controlled trial (RCT), the “Catheter Ablation vs. Standard Conventional Therapy in Patients with Left Ventricular Dysfunction and Atrial Fibrillation” (CASTLE-AF) RCT, subgroup analyses of the “Catheter Ablation vs. Antiarrhythmic Drug Therapy for Atrial Fibrillation” (CABANA) RCT and recent pooled analyses of randomized data and meta-analyses demonstrated a reduced all-cause mortality in heart failure patients undergoing CA for AF compared to the medical AF treatment (24–29). However, these collectives were on average about 10 years younger, involved fewer patients in NYHA functional class III/IV than the present study, and, obviously, excluded patients with high-grade MR. In addition to prognostic efficacy, the safety of the procedure is also a critical consideration. In the present study no major complications or periprocedural interferences with the TEER device associated with CA were observed. Also, a case series limited to 14 patients with CA of AF after TEER reported no major complications except for a transient ischemic attack which proved to be completely reversible in a patient on vitamin-k antagonists two days postprocedurally (30). The complication rate reported in the landmark studies cited above is also encouragingly low, at approximately 2%, with the vast majority being access related bleeding complications and percutaneously manageable pericardial effusions (24–26). Here, however, elderly patients such as those in the present collective are not well represented. “Real-world” data derived from observational studies and meta-analyses suggest that patients over 75 years of age experience similar to slightly higher complication rates, again mainly due to non-fatal bleeding at the access site and pericardium rather than systemic or cerebral emboli (31–34). Considering this low rate of expectable major complications associated with CA, the present sample size is insufficient to reliably assess the safety of interventional rhythm control in this collective. Overall, however, it can be stated that CA for AF performed in an experienced and high-volume center can be considered safe even in elderly and multimorbid patients, and most likely applies to the TEER collective as well.

## Limitations

Given the nature of an observational cohort study, the results cannot demonstrate a causal relationship.

Despite careful adjustment for baseline differences by the two established and independent statistical procedures, PSM and multivariable Cox regression, the possibility of residual bias remains. Thus, despite PSM, it was not possible to fully balance the type of TEER device used between the CA and PT groups, as evidenced by the more frequent use of the PASCAL device in the CA group compared with the PT group. An impact of this imbalance on the primary endpoint all-cause mortality however, is unlikely, as both devices do not differ in their basic mode of operation and as no relevant differences in clinical efficacy or safety were observed between PASCAL and the MitraClip device in the randomized controlled Edwards PASCAL Transcatheter Valve Repair System Pivotal Clinical (CLASP-IID) Trial, which was also confirmed by our data (35).

Due to the design of the present study, however, it is theoretically possible that in spite of careful adjustment for the comprehensively recorded clinical and procedural characteristics, there is a bias in the selection (selection bias) of the individual cohorts analyzed.

Compared with clinical trials, the proportion of missing data, although small, and the use of a registry may limit precision, which may limit internal validity. In addition, we cannot evaluate the efficacy of AF therapies in terms of sinus rhythm stability or individual AF burden, respectively, and no detailed information on incidence of rehospitalizations or on individual causes of death during the follow-up period because the relevant data were not fully available. Nevertheless, clinically highly relevant hard end points were addressed.

## Conclusion

Due to intimately shared pathophysiological mechanisms, MR, HF, and AF constitute a highly prevalent and yet equally fatal trio that continues to dramatically darken a patient's prognosis even after successful TEER. In the present study, we demonstrate with PSM and Cox regression that CA of concomitant AF is associated with a significantly better long-term outcome compared to pharmacological therapy and may even reverse the evident prognostic disadvantage of AF in the TEER collective.

## Clinical perspectives

In light of the growing evidence of prognostic benefit and safety of CA of AF in the overall cohort of HF patients, our data, albeit not randomized, provide strong evidence for interventional rhythm control of AF as an essential part of a holistic management of TEER patients. We point out the importance of treating concomitant AF, as this is a very promising approach to improve the prognosis of this unique and continually growing HF population. Prospective randomized confirmation of the present results and further investigation of procedural aspects of CA in a larger TEER collective are strongly encouraged.

## Data Availability

The raw data supporting the conclusion of this article are available upon request.
