# Supplementary material for: Catheter ablation of concomitant atrial fibrillation improves survival of patients undergoing transcatheter edge-to-edge mitral valve repair
Source: Front Cardiovasc Med. 2023 Aug 14;10:1229651. doi: 10.3389/fcvm.2023.1229651 (PMC10461005; doi:10.3389/fcvm.2023.1229651)
Supplement: Supplementary file 1 [file Table1.docx]

**Table S1:** **Comparison of clinical and procedural characteristics of patients lost to follow-up in comparison with those of patients not lost to follow-up.**

|  |  |  |  |
| --- | --- | --- | --- |
| Variable | Patients not lost to FU  (n=766) | Patients lost to FU  (n=55) | p-value |
| Age (years) | 78 ± 8 | 79 ± 7 | 0.1 |
| euroSCORE II (%)* | 16.1% (20.8) | 12.4% (10.7) | 0.3 |
| STS Risk Score (%)* | 6.7% (8.4) | 5.8% (5.2) | 0.1 |
| Male sex | 61.9% (474) | 72.7% (40) | 0.1 |
| NYHA class I  NYHA class II  NYHA class III  NYHA class IV | 0.1% (1)  3.4% (26)  75.8% (581)  20.6% (158) | 0% (0)  0% (0)  72.7% (40)  27.3% (15) | 0.3 |
| COPD | 18.3% (140) | 10.9% (6) | 0.2 |
| CAD | 62.4% (478) | 60% (33) | 0.8 |
| Prior CAB-OP | 28.5% (218) | 18.2% (10) | 0.1 |
| Prior PCI | 53.9% (413) | 54.5% (30) | 1 |
| Pre-existing ICD | 22.5% (172) | 20% (11) | 0.7 |
| Pacemaker  *CRT* | 29.6% (227)  *14.4% (110)* | 27.3% (15)  *14.5% (8)* | 0.8  *1* |
| Diabetes mellitus | 30.4% (233) | 21.8% (12) | 0.2 |
| Arterial hypertension | 81.5% (624) | 74.5% (41) | 0.2 |
| Prior Stroke | 9.4% (72) | 14.5% (8) | 0.2 |
| LVEF ≥50%  LVEF 41-49%  LVEF ≤40% | 38.8% (297)  11.4% (87)  49.9% (382) | 45.5% (25)  14.5% (8)  40% (22) | 0.3 |
| Atrial fibrillation | 73.9% (566) | 76.4% (42) | 0.8 |
| GFR (mL/Min) | 50 ± 26 | 53 ± 19 | 0.3 |
| NT-proBNP (ng/L)* | 2299 (4923) | 2050 (5234) | 0.6 |
| AF patients after PVI | 5.9% (45) | 5.4% (3) | 0.8 |
| TR grade III | 17.9% (137) | 29.1% (16) | 0.06 |
| Degenerative MR etiology  Functional MR etiology  Mixed MR etiology | 36.9% (278)  52.5% (402)  11.2% (86) | 27.3% (15)  54.5% (30)  18.2% (10) | 0.1 |
| Median procedure duration (min)* | 82 (62) | 68 (34) | 0.1 |
| Number of clips implanted* | 1 (1) | 1 (0) | 0.3 |
| Periprocedual MR reduction  (carpentier grade) | Δ2.0 ± 0.6 | Δ2.0 ± 0.3 | 0.6 |
| Length of hospital stay (days)* | 7 (5) | 5 (4) | 0.1 |
| Overall-MACCE  *Cerebral/systemic thromboembolic event*  *Bleeding requiring intervention*  *In-hospital death from cardiovasc. cause* | 5.5% (42)  *0.7% (5)*  *3.3% (25)*  *2.2% (17)* | 3.6% (2)  *0% (0)*  *1.8% (1)*  *1.8% (1)* | 0.8  *1*  *1*  *1* |
| In-hospital death from any cause | 3.7% (28) | 1.8% (1) | 0.7 |
| Heart Failure Therapy |  |  |  |
| ACE-/AT1 Inhibitors | 72.7% (557) | 65.5% (36) | 0.3 |
| ARN Inhibitor | 13.1% (100) | 20% (11) | 0.2 |
| Beta Blockers | 89.2% (683) | 83.6% (46) | 0.3 |
| Diuretics | 93.1% (713) | 89.1% (49) | 0.5 |
| Aldosteron antagonists | 48.4% (371) | 45.5% (25) | 0.7 |
| SGLT-II-Inhibitors | 4.6% (35) | 7.3% (4) | 0.5 |
| Vericiguat | 0% (0) | 1.8% (1) | 0.07 |

**Median + IQR*

*ACE, Angiotensin-converting enzyme; AF, atrial fibrillation; ARN; angiotensin-neprilysin; AT1, angiotensin 1; CAB-OP, coronary-artery-bypass OP; CAD, coronary artery disease; COPD, chronic obstructive pulmonary disease; CRT, cardiac resynchronization therapy; FU, follow-up; GFR, glomerular filtration rate; ICD, implantable cardioverter defibrillator; LVEF, left ventricular ejection fraction; MACCE, Major Adversive Cardiovascular and Cerebrovascular Events; MR, mitral valve regurgitation; NYHA, New-York-Heart-Association; PVI, pulmonary vein isolation; PCI, percutaneous coronary intervention; TR, tricuspid valve regurgitation*
